# Health technology assessment of new retinal treatments; the need to capture healthcare capacity issues

**DOI:** 10.1038/s41433-022-02149-5

**Published:** 2022-06-28

**Authors:** Richard Gale, Oliver Cox, Craig Keenan, Usha Chakravarthy

**Affiliations:** 1 Hull York Medical School, University of York, York and Scarborough Teaching Hospitals NHS Foundation Trust, York, UK; 2 Global Access, F. Hoffmann-La Roche, Basel, Switzerland; 3 PHMR LTD, Westport, Ireland; 4 Queen’s University of Belfast, Royal Victoria Hospital, Belfast, Ireland

**Keywords:** Health care economics, Macular degeneration

The increasing prevalence of key retinal conditions [1–5] and increasing utilisation of resource intensive treatments for these conditions [6–8], have led to concerns regarding the current and future resource burden on retinal services [9]. Any potential capacity shortfalls threaten the delivery of optimal care to patients and may have a negative effect on clinical outcomes [10, 11]. As a result, approaches to increase the efficiency with which intravitreal injection clinics are run have been proposed and implemented [11]. While strategies such as these can provide gains in capacity, they are unlikely to be able to completely address the capacity issues. Innovation in ophthalmologic care, including the development of newer, more durable treatment options are therefore likely to represent a key approach to mitigating these capacity issues.

In many health systems, decisions regarding patient access to new treatments are typically based on an evaluation of their value relative to the existing standard(s) of care. Value, in such cases, can be defined as the *‘improvement in the quality and/or length of life and/or financial value gain, defined as cost-effectiveness and the financial return on investment for the direct medical costs expended*’ [12]. To date, these evaluations have generally only considered budgetary constraints on the health system in measuring value, ignoring resource constraints, or capturing them qualitatively outside the main quantitative evaluation. While this approach is arguably appropriate, it can lead to inaccurate evaluations where capacity constraints exist [13, 14].

From an ophthalmology perspective, economic evaluations which only focus only on budgetary constraints would capture any reduced resource burden associated with new treatments purely in terms of the impact on costs. While these cost savings are an important and relevant consideration, in health systems with significant ophthalmic capacity constraints it may be particularly important to also consider capacity constraints, and their impact on patients, health systems and society, when assessing the value of new treatments. That is, more durable treatments should mitigate the need for treatment to be delayed or missed due to shortfalls in capacity thereby improving patient outcomes. Importantly, given the substantial impact of blindness on quality of life, the high value patients place on avoiding blindness, and the significant healthcare and societal costs associated with vision loss and blindness, these improvements in patient outcomes are likely to be valued more highly than any cost savings due to the reduction in resource use [15, 16]. A failure to capture the capacity issue when assessing newer more durable treatments may therefore undervalue their overall benefits.

In order to better demonstrate this issue, we have broadly conceptualised these alternative perspectives in a simple hypothetical example presented in Fig. 1. In the first panel of the Figure (Fig. 1A), we demonstrate a standard, budget-constrained approach under which the orange shaded area represents the number of avoided injections due to the use of a new treatment with lower treatment frequency than standard of care. In this approach one would attach costs to both the current and new treatments and the reduced number of injections associated with the new treatment would be captured when calculating the difference in costs between the treatments (net cost). By combining this with data on patients’ outcomes under both treatments one would determine the relative or incremental value of the new treatment. In the second panel (Fig. 1B) we demonstrate an approach which includes a capacity perspective, where the orange shaded area again represents the number of avoided injections due to the use of a new treatment and where the blue shaded area represents the number of required injections that might be foregone with standard of care due to a capacity ceiling being reached. The orange shaded area would again be captured in the calculation of net costs but in this case the blue shaded area would be captured as a difference in patient outcomes and resultant impact on longer term healthcare and societal costs. That is, using randomised trial and/or real-world data one can translate the foregone injections into service disruptions and a resultant increase in vision loss. Service disruptions during the Covid-19 pandemic have provided an ideal opportunity for the generation of evidence of this nature, with several studies demonstrating greater visions loss among nAMD, DME and RVO patients who experience delays than those who did not [17]. Notably, Greenlee et al. found that among nAMD patients who experienced treatment lapses of ≥3 months the deficits in visual acuity did not improve following resumption of treatment [18]. As noted above, these poorer outcomes are likely to have substantial impact on the quality of life of patients and their management is likely to lead to increased future costs to the healthcare system and society as whole. As such, it would appear that more durable treatments are likely be undervalued if capacity constraints are not accurately reflected in economic evaluations.

**Fig. 1:**
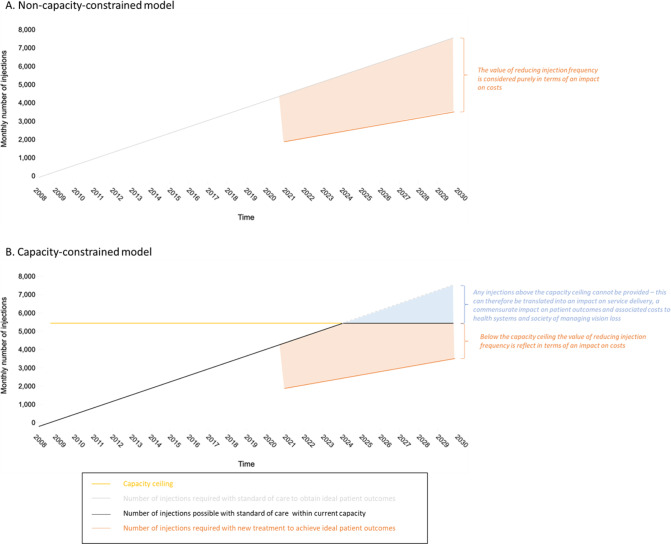
Illustrative example of the impact of a capacity ceiling of 5500 injections per month on the delivery of standard of care and on delivery of a new treatment which halves the required number of injections. The orange shaded areas represent the potential cost savings associated with avoided injections that might be realised with a hypothetical new treatment while the blue shaded area represents the potential number of necessary injections that would go unadministered due to a capacity ceiling, which could in turn be extrapolated to a deficit in patient outcomes. Numbers underlying example based roughly on actual and forecasted number of injections in a UK tertiary hospital setting as presented in ref. [9].

We have used a simple example here in which we have conceptualised the impact of new treatments on resource use only in terms of reduced injection burden and in which capacity is viewed in terms of a simple capacity ceiling. In practice, models which fully capture capacity issues will need to be more complex in nature, reflecting additional aspects of patient care and the true complexities of resource use and capacity constraints. While the model presented is simple and hypothetical in nature, we believe it could help in the conceptualisation of more detailed and complex systems. Despite their added complexity, we believe the development of such models is warranted, particularly for health systems with significant ophthalmic capacity constraints, as they should allow for more accurate economic evaluation of the value of new treatments and better support decision-making around reimbursement and patient access for these treatments.

## Conclusions

Emerging therapies associated with more durable effects and reduced resource use can help to address these capacity issues, however common approaches to economic evaluation of new therapies may not adequately capture these capacity-related benefits. Further work is needed to ensure capacity-related benefits are adequately captured and considered when evaluating new treatments for retinal disease; by doing so we can optimise patient access to retinal treatments and ultimately improve patient outcomes.
